# Detection, quantification, and characterization of polystyrene microplastics and adsorbed bisphenol A contaminant using electroanalytical techniques

**DOI:** 10.1007/s00604-023-05780-5

**Published:** 2023-05-09

**Authors:** Juan C. Vidal, Javier Midón, Ana B. Vidal, Dragos Ciomaga, Francisco Laborda

**Affiliations:** Group of Analytical Spectroscopy and Sensors (GEAS), Institute of Environmental Sciences (IUCA), University of Zaragoza, C/ Pedro Cerbuna 12, 50009 Zaragoza, Spain

**Keywords:** Polystyrene microparticles, Chronoamperometry, Blocking nanoimpact electrochemistry, Electrochemical impedance spectroscopy, Adsorptive enrichment

## Abstract

The potential applications of electroanalytical techniques for the quantification and size characterization of nonelectroactive polystyrene microplastics is reported, in addition to characterizing the kinetics of adsorption of bisphenol A on these polystyrene microparticles. The individual adsorption events of very diluted polystyrene microparticles dispersions on glassy-carbon microelectrodes produce the blocking of the charge transfer of a mediator (ferrocene-methanol) thus decreasing the current of the recorded chronoamperogram in a stepwise manner. The magnitude of the current steps are in the order of pA values and can be related to the diameter of the plastic microparticles in the size range 0.1 to 10 µm. The frequency of the current steps in the domain time used (120 s) allows to quantify the number concentration of these microparticles in the range 0.005 to 0.500 pM. Electrochemical impedance spectroscopy confirms the adsorption of the polystyrene microplastics on carbon microelectrodes (and to a lesser extent on platinum microelectrodes) under the same experimental conditions as above. On the other hand, the adsorbed microplastics become concentrators of other pollutants found in the environment. The sensitive differential-pulse voltammetry determination of bisphenol A (linear range 0.80–15.00 µM; detection limit 0.24 µM) was used together with a simple separation procedure for studying the adsorption of bisphenol A on polystyrene microparticles. The adsorption capacity (mg of bisphenol A retained per g of the polystyrene microplastics) decreased from approximately 5.7 to 0.8 mg g^−1^ with increasing dosages of polystyrene microparticles from 0.2 to 1.6 g l^−1^. The adsorption isotherms were modeled resulting in a monolayer of bisphenol A adsorbed on the microplastics (i.e., best fitted to a Langmuir model).

**Graphical abstract:**

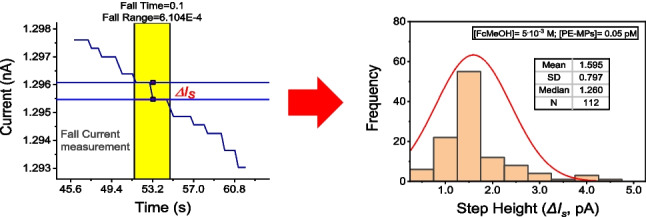

**Supplementary information:**

The online version contains supplementary material available at 10.1007/s00604-023-05780-5.

## Introduction

Microplastics (MPs) are particles from the physical and chemical degradation in the environment of plastics pollutants, having a typical size in the range of 0.1–5000 μm. The global production of plastics is estimated to exceed 370 million tons each year, 68 million tons in Europe in 2020, and is expected to double in the next 30 years [1]. Consequently, the global production of plastics may exceed 100 million metric tons per year globally in the year 2050. Large plastics, in turn, degrade in the environment due to climatic conditions (e.g., UV photodegradation, hydrolysis, mechanical abrasion, biodegradation), producing microplastics (diameters about 5 mm–100 nm) and even nanoplastics (< 100 nm), thus increasing their persistence, mobility, ubiquity, occurrence, toxic potential and capacity to retain other pollutants.

Microplastics have the ability to adsorb on their surface a vast number of contaminants present in the environment, for example, heavy metals, persistent organic compounds and pharmaceuticals, particularly endocrine disruptors and antibiotics [2]. Sorption of pollutants onto MPs depends on the physicochemical properties of both the sorbate and the sorbent, besides the medium properties [2]. Consequently, numerous studies report the characterization of the MPs adsorption processes using a wide variety of procedures and analytical techniques, for example, by using ultrafiltration [3] and high performance liquid chromatography coupled to mass spectrometry [4].

Electroanalytical techniques provide useful chemical-physical information in the study of micro and nanomaterials or nanoparticles [5–8], but have not been used together for pollutants adsorbed on them. Faradaic particle-electrode impact electrochemistry has been used to study polyethylene (PE) microparticles suspended in an aqueous solution using carbon-fiber microelectrodes, which allows identification, particle size distribution, and concentrations for microparticles of sizes 1–10 µm [9]. There is no posibility of direct faradaic electron transfer from the polyethylene particles themselves due to the fact that PE is an insulator, but cathodic current spikes are due to solubility of electroactive oxygen in PE microparticles.

MPs are intrinsically electroinactive, so charge transfer mediators are commonly used for indirect measurements of electrical currents related to these MPs as a function of applied potential. The first report of time-resolved electrochemical detection of discrete adsorption events appeared in 2004. The adsorption of individual electroinactive micron-sized carboxylated latex beads on gold microdisk electrodes (2.5 µm diameter) produces steplikes decreases in current ($\Delta {\varvec{i}}$), namely, blocking collisions corresponding to the blockage of the flux of mass transfer of a redox mediator (FcMeOH, ferrocene methanol) [10, 11]. A theoretical model estimates the magnitude of the current steps due to blocking collisions proportional to the square of the particle size (*r*_*p*_^2^), the concentration of the redox mediator (*C*_*m*_), and inversely proportional to the size of the working microelectrode (*r*_*electr*_), which was corroborated experimentally with 1 µm polystyrene microparticles (PS-MPs) on Pt microelectrodes [12]. In these previous reports on blocking collision experiments, the main objective is the theoretical evaluation of the diameter of the microparticles under a physical point of view, but not the analytical calculation of the concentration of these microparticles.

The feasibility of detecting electrochemically inactive single biomacromolecules (enzymes, antibodies, DNA) and polystyrene (PS) nanospheres by blocking electrochemistry has been demonstrated by Bard´s group [13]. By oxidizing large concentrations of ferrocene methanol, time-resolved discrete adsorption events from electrochemical collision experiments can be differentiated from the background. Chronoamperometric current step magnitudes and collision frequencies can be related to the size and microparticle concentrations, respectively. In a different approach, the time of first arrival by migration in blocking collision experiments allows measurements of ultralow (sub-femtomolar) concentration of electroinactive analytes (PS beads) dispersed in solution [14]. The individual adsorption events of sub-µM silica and PS spheres (310–530 nm diameter) have been also detected by monitoring the blocking of redox mediator diffusion to Pt ultramicroelectrodes substrates [14].

Time-resolved inductively coupled plasma mass spectrometry (ICP-MS) has been succesfully applied in our research group to the detection of plastic microparticle by the so-called single particle ICP-MS [15, 16]. Despite the inherent limitations of carbon detection by ICP-MS, Laborda et al. developed and validated an analytical method for detection of plastic microparticles over ca. 1 µm and 100 particles per mL, being applied to the screening of microplastics in personal care products and released from food packagings [16].

The aim of this work is the use of various electroanalysis techniques that, synergistically, allow the characterization and quantification of PS-MPs as well as studying their interaction with BPA. Blocking particle-collision chronoamperometry, electrochemical impedance spectroscopy (EIS), and differential-pulse voltammetry (DPV) were used for this purpose.

BPA (4,4′-(propane-2,2-diyl)diphenol) was taken as a model of environmental pollutant strongly adsorbed by PS-MPs. It is a potent endocrine-disrupting compound with a chemical structure analogs to endocrine hormones (e.g. estradiol, diethyl-stilbestrol) due to phenol groups, so it has affinity to bind estrogen receptors [17]. Electrochemical sensors and biosensors play an important role in the very sensitive quantification of BPA, due to the well-known electroactivity of these phenolic groups present in the molecule [18].

We report the particle collision quantification and size-characterization of nonconducting nonelectroactive plastic microparticles. Collisions of the PS-MPs results in the effective decrease in the electroactive area of a working carbon microelectrode and a decrease (blocking) in the current of a charge transfer mediator added to these dispersions. EIS measurements confirms the effective adsorption of dispersed PS-MPs on micro glassy-carbon working electrodes (µGCEs). The DPV (differential-pulse voltammetry) quantification of BPA in solutions after being in contact with dispersed microplastics allows to study the kinetics and models for the adsorption of this contaminant on PS-MPs.

## Experimental

### Reagents

Bisphenol A (Alfa-Aesar, ref. A10324, analytical grade 97% quality). Standard 2^.^10^–3^ M stock solutions of BPA were prepared in pure ethanol. Solutions of KCl (Sigma-Aldrich, Merck KGaA, Darmstadt, Germany) were used as supporting electrolytes. Solutions of ferrocene-methanol (FcMeOH) (Apollo Scientific, ref.1273865, analytical grade) were used as electrochemical charge-transfer mediator. The polystyrene monodispersed microparticles were aqueous dispersions certified as analytical standard of size and concentration (Sigma-Aldrich, Merck KGaA, Darmstadt, Germany). Nominal diameters (Ø) of 10 µm (ref. 72,822), 0.5 µm (ref. 95,585) and 0.10 µm (ref. 90,517) were used. Microsized polystyrene (PS) and expanded polystyrene (EPS) were prepared from commercial plastics by sanding and later characterized by optical microscopy (Carl Zeiss, Axio Imager A2, reflected light optical microscope with polarized light).

### Instrumentation

Electrochemical measurements were carried out with: (i) μAutolab-III potentiostat (Metrohm (± 1nA, ± 0.1 mV) for cyclic and pulse voltammetry measurements. Equipped with a FRA2 module for electrochemical impedance measurements at frequencies between 10 kHz and 10 μHz; (ii) Eco-Chemie Autolab PGSTAT10 potentiostat (Methrom) equipped with a low current module (ECD) for nanoimpact measurements (currents in the order of pA-fA). The microelectrodes were protected inside a Faraday cage and connectig cables were specially shielded to avoid external noise. The ECD module (Metrohm) adds two very low current measurement scales (1 nA and 100 pA), being able of measuring currents up to 0.30 fA, and incorporating Sallen-Key noise filters with 3 RC time constants. The data acquisition rate for the chronoamperometric measurements was 20 Hz. Metrohm’s own control software NOVA v.2.1.2 was used in both potentiostats. This software was used for the voltammetric measurements and fittings to obtain from the measured impedance data the individual component magnitudes of the appropiately chosen equivalent circuits.

The following voltammetric electrodes were from BAS-i-BioAnalytical Systems Inc. (West Lafayette-IN, USA): Carbon fiber microelectrode (μGCE), (diameter 11 ± 2 μm, ref. MF-2007); Platinum microelectrode (μPtE), diameter 10 ± 1 μm (ref. MF-2005); Glassy carbon electrode (mini) (GCE) (diameter Ø ~ 3 mm (ref. MF-201); Ag/AgCl (mini) reference electrode (ref. MF-2079); Platinum wire auxiliary (mini) electrode (ref. MW-4130).

For separations in eppendorf polypropylene tubes of PS-MPs from the BPA solutions a Heareus multifuge X1R centrifuge (Thermo Scientific, 41223274) was used. Size characterization of the prepared microplastics (PS, EPS) were carried out with an optical microscope of reflected light Axio Imager A2 (Carl Zeiss) with polarized light. A Reax 02 orbital shaker (Heidolph, Ref. 200473492) was used for MPs-BPA adsorption studies.

**Fig. 1:**
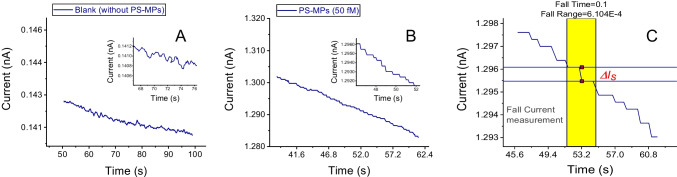
Typical changes in the steady-state currents of the chronoamperograms measured in **A** a blank solution (FcMeOH 5^.^10^–3^ M in 1^.^10^–3^ M KCl); **B** the same blank solution as above in the presence of PS-MPs 0.050 pM. Working electrode: µGCE. **C** Magnified view of a discrete current step measurement (∆*I*_*s*_)

For the evaluation, counting and quantification of the step current magnitudes from the recorded chronoamperograms and fittings of data the software OriginPro 2020 (v.9.7.0.188, OriginLab Corp., Northampton, MA, USA, www.originlab.com) was used. Two main tools were used within this program. The signal processing tool called “Smooth” was used in the “Percentile Filter” mode to eliminate noise from the measurement and to distinguish the step currents due to collisions. The “Rise Time” gadget tool was used to measure the height of the current steps. The way current steps were measured is illustrated in Fig. 1C.

### Nanoimpact electrochemical size-characterization and quantification of polystyrene microparticles

A PGSTAT10 potentiostat equipped with an ECD low-current measurement module was used. The electrochemical cell was isolated inside a faradaic cell for avoiding external noise, and all the coaxial cable connections with the potentiostat shielded with aluminum foil. A deep cleaning of all the materials was necessary to avoid the presence of dust or other microparticles in suspension. The working µGCE was previously polished with alumina (Ø ~ 0.05 µm), rinsed with deionized water, sonicated in water for three minutes and rinsed again with deionized water, as previously reported [8]. Once the supporting electrolyte (KCl, 1^.^10^–3^ M) and mediator (FcMeOH, 2.5^.^10^–3^ M) blank solutions, were prepared in the voltammetric cuvette, the polystyrene aqueous suspension was added to obtain a concentration in the cuvette in the range 0.005–0.500 pM, at the same time that agitating with nitrogen flow for the rapid convective homogenization in a controlled time of 5.0 s at an applied potential of *E*_*a*_ = 0.0 V. The chronoamperomagram was immediately recorded by applying a potential of *E*_*a*_ =  + 0.50 V, about 0.20 V positive of the half-wave potential for the FcMeOH oxidation, for 150 s. The current oxidation of FcMeOH is insensitive to oxygen and nitrogen. The current-time discrete steps due to the adsorption of the microparticles blocking the charge-transfer of the mediator were counted from the recorded chronoamperograms between 10 (without taking into account the initial capacitative current) and 130 s.

### Impedance characterization of the adsorption of PS-MPs on GCE electrodes

Electrochemical impedance measurements were carried out with a *μAutolab-III potentiostat*, *equipped with a* FRA2 impedance response analyzer module in a frequency range of 10 kHz to 10 Hz, at a constant potential (*E*_*e*_ =  +*0.5 V*) and with a maximum variation of the sinusoidal amplitude of *∆E*_*e*_ = 5 mV. Impedance measurements were plotted on Nyquist graphs (variation of the imaginary part of the impedance versus the real part), and Blode graphs (variation of the impedance as a function of frequency) before choosing the most appropiate equivalent circuit fitting the real electrochemical interfases. Two experimental impedance measurement were performed, both witg µGCE and µPtE working electrodes:

- Capacitive type measurements ([KCl] = 1^.^10^–3^ M; [PS-MPs] = 0.050 pM). For this, charge-transfer mediator was not used to enhance the impedance due to the double electrical layer of the electrode, in evaluating changes due to the PS-MPs adsorbed on µGCEs. The series RC equivalent circuit of the Fig. 3A was the most appropiate.
- Faradaic measurements with 2.5^.^10^–3^ M FcMeOH mediator ([KCl] = 1^.^10^–3^ M; [PS-MPs] = 0.050 pM). In this case the impedance of the charge transfer of the mediator (FcMeOH) as a function of the polystyrene adsorption time is enhanced. The parallel circuit of the Fig. 3B was used for the fittings of this kind of measurements.

### Differential-pulse voltammetry study of the kinetics of adsorption of bisphenol A on polystyrene microplastics

PS and EPS microparticles were obtained from plastic objects of daily use by sanding with sandpaper of grain sizes numbering 320 for PS and 400 for EPS. The size of the microplastics was characterized by optical microscopy in small representative samples from the total obtained. Average sizes and standard deviations (SD) were calculated by measuring over 150 nanoparticles in random regions of the images. The image analysis and size determinations were carried out using ImageJ software (version 1.52), obtaining size distributions with mean diameters around 40 and 80 μm for PS and EPS MPs, respectively.

Dispersions of the PS-MPs (in aliquot concentrations from 0.2 to 1.6 mg L^−1^) were mixed with BPA solutions (final concentration 1.2 10^–4^ M) maintaining orbital agitation between 0.00 and 24.00 h in eppendorf tubes. In order to calculate the kinetics of adsorption of BPA on the MPs, in controlled times (between 0.00 and 24.00 h), the dispersed MPs were separated by centrifuging at 7500 rpm for 10 min and an aliquot of the solution (200 µL) was quickly taken for the voltammetric determination of the dissolved BPA. In this way, the concentration of BPA not adsorbed by the MPs was measured, and by difference the amount of BPA adsorbed per unit mass of the MPs. Control measurements of BPA in the absence of MPs showed that there were no significant losses in BPA concentration after the centrifugation process.

To quantify the concentration of BPA in solution, differential-pulse voltammetry (DPV, *ΔE*_*p*_ = 25 mV; *ΔE*_*s*_ = 5 mV; v = 6 mV s^−1^) was used with potential scanings from + 0.45 to + 0.95 V (to minimize the formation of polyphenols at more anodic potentials). BPA linear calibrations were previously made between concentrations from 0.20 to 15.00 µM of BPA (0.05–3.42 µg mL^−1^). Bare GCE working electrodes and KCl 0.10 M as supporting electrolyte were used in all determinations. Since electrochemically formed polyphenols adhere strongly to GCE, the working electrode was polished and washed after each voltammetric measurement. After washing with water, the GCE was polished with alumina oxide (0.05 µm diameter) by rubbing on a felt cloth for about 2 min. Finally, the electrode was washed with distilled water to remove any traces of alumina.

## Results and discussion

### Blocking detection, quantification and size-characterization of PS-MPs

The most common way to detect nanoparticles in electrochemical collision techniques is based on electroactive nanoparticles. Starting from very dilute solutions and using (ultra)microelectrodes, discrete current increases (spikes) are measured in the *i-t* curves (chronocoulometry) by applying the appropriate potential [6, 19]. In more recent developments, electrocatalytic amplification, emulsion droplets, single bioparticles, photoelectrocatalysis, and blocking strategies have emerged [20].

We have previously studied the characterization (10–100 nm size distributions) and quantification of silver nanoparticles (AgNPs) with a particle collision chronocoulometry (PCC) method [6]. In very diluted AgNPs suspensions, individual electroactive nanoparticles stochastically impact the surface of a carbon microelectrode at an anodic potential and are completely oxidized, resulting in small current transients (spikes). The charge passed during the spikes are quantitatively linked to the size of each nanoparticle, therefore extracting size distributions from the spike transients [6].

In this study, a different approach is used for the electroinactive plastics. PS-MPs strongly adsorb on carbon electrodes, as it was demonstrated with electrochemical impedance spectroscopy (the “Impedance characterization of the adsorption of PS-MPs on carbon and Pt electrodes” section). Upon impacting the individual PS-MPs from very diluted dispersions, predominantly by electrophoretic migration (in conditions of low concentrations of supporting electrolyte), they are retained (adsorbed) on the surface of the working electrode. By using FcMeOH as a charge-transfer mediator, discrete decreasing steps in steady-state current of the recorded chronoamperograms are observed in very diluted dispersions of the PS-MPs (Fig. 1B). These stepped transients recorded in the steady-state current are due to the individual collision an irreversible adsorption (sticking) of the microparticles on the µGCE, causing the blocking of the charge-transfer of the mediator. In blank solutions, without the plastic microplastics, no discrete steep current decreases were observed on the recorded chronoamperograms under the same experimental conditions (Fig. 1A). The residual random noise in the blank solutions was of the order of ≤ 0.23 pA peak-to-peak (Fig. 1A), while the steps of the currents decay (*∆I*_*s*_) were of the order of + 0.6–1.2 pA for a solution of PS-MPs (nominal diameter of 0.50 µm) 0.050 pM (Fig. 1B).

The frequency of the current steps under the diffusion limited oxidation of the mediator strongly depends on the concentrations of the supporting electrolyte (KCl), in agreement with other authors [10, 11]. By using KCl 1^.^10^–3^ M, a dispersion of 0.050 pM of PS-MPs gave a frequency of steped down currents of 0.92 Hz in the range 10 to 130 s. Nevertheless, with a supporting electrolyte concentration of KCl 5^.^10^–3^ M the frequency of the steps decreased to about 0.28 Hz. Additionally, decreasing the applied potential from + 0.5 to + 0.3 V decreased significatively the frequency of the steps to about 0.09 Hz for the same number of PS-MPs (KCl 1^.^10^–3^ M). This demonstrates that the magnitude of the electric field set by the working electrode potential is responsable of the migration of the PS-MPs.

In conclusion, mass transfer of the PS-MPs under conditions of very low concentrations of KCl (milimolar order) is mainly due to electrophoretic migration of the negatively charged PS-MPs, rather than their low free diffusion mass transfer rate or brownian motion. For example, a theoretical calculation of the frequency of the steps by the Stokes-Einstein equation in the diffusion of spherical PS microparticles (310 nm diameter) predicts by diffusive flux alone a much lower sphere arrival frequency of only 1.7^.^10^–4^ Hz by using a 2 µm Pt microelectrode, which demonstrates that spheres do not move to the microelectrode by diffusion alone and migration is the dominant mode of the transport of the PS microparticles [11]. The same authors conclude that electric field established with low-electrolyte concentrations give rise to over 3 orders of magnitude increase in the flux of charged microparticles [10].

A theoretical model estimating the average value of the current step magnitude in blocking collisions establishes a proportionality between the height of steps (*∆I*_*s*_) to the the square of the radius of the particless (${r}_{p}^{2}$), the concentration of the mediator (*C*_*m*_), and inversely proportional to the radius of the microelectrode (*r*_*electr*_), resulting in equation: $\Delta$*I*_*s*_ $\propto\frac{r_p^2\;\cdot{\;C}_m}{r_{electr}}$ [12]. Consequently, the magnitud of *∆I*_*s*_ is related to the size of the microparticles that produce the blocking collisions. One of the objectives of this report was to explore the analytical use of the electrochemical blocking collisions measurements both for the characterization of the size of PS-MPs and to quantify the number of these microparticles.

**Fig. 2:**
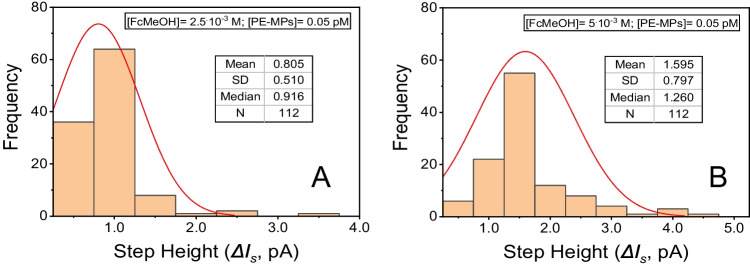
Distributions of the current step values counted between 10 and 130 s. from current–time chronoamperograms of the mediator FcMeOH in concentrations of the following: **A** 2.5^.^10^–3^ M and **B** 5^.^10^–3^ M. In both cases, the concentration of PS-MPs (0.50 µm diameter) was 0.050 pM. Supporting electrolyte: KCl 1^.^10^–3^ M. The µGCE was held to a potential of E_a_ =  + 0.50 V. vs Ag/AgCl

According to the previous relationship, higher concentrations of the mediator improve the sensitivity of the collision detection (ie., increase the magnitude of *∆I*_*s*_ values). Higher concentrations of the FcMeOH directly increase the flux of the mediator to the electrode and then will increase proportionally the magnitude of the flux blockage of the current steps. It was supported by measuring 0.050 pM of PS-MPs (0.50 µm diameter) with concentrations of the FcMeOH mediator of 5^.^10^–3^ and 2.5^.^10^–3^ M. The resulted distributions of the frequency vs step heights (*∆I*_*s*_) are given in Fig. 2. The mean value of the *∆I*_*s*_ for a concentration 5^.^10^–3^ M of the mediator is approximately twice (1.98 times) the same value for a concentration 5^.^10^–3^ M in accordance to the above equation.

From calibrations using size standards of PS-MPs, the distributions of *∆I*_*s*_ (mean ± sd in pA units) can be used to estimate size distributions of unknown samples. This was supported by comparing the results of PS-MPs of 0.5 µm diameter with those of 0.1 µm diameter. The results are given in the supporting information (Fig. SI-2).

Experiments with platinum working microelectrodes (µPtEs) produced lower sensitivities (lower *∆I*_*s*_ values) under the same experimental conditions as with the µGCEs, probably due to a lower rate of charge transfer in the oxidation of the mediator in the platinum substrate than compared to the glassy-carbon (Fig. SI-3 of the supporting information). EIS measurements also confirmed that µPtEs have lower adsorption capacity than µGCEs of the PS-MPs (see “the Impedance characterization of the adsorption of PS-MPs on carbon and Pt electrodes” section).

Another interesting conclusion is the analytical application of the procedure to quantify the number of polystyrene nanoparticles in dispersion, given the proportional relationship with the frequency of the individual adsorption events. The graphical representation of the frequency of the collisions of the PS-MPs (0.5 µm diameter) as a function of the logarithm of the concentration of the microparticles gave rise to a linear relationship in the range 0.005–0.500 pM (equation: y = 17.43^.^ log [PS-MPs, pM] + 137.5, *R* = 0.9984). See Fig. SI-4 of the supporting information.

## Impedance characterization of the adsorption of PS-MPs on carbon and Pt electrodes

Electrochemical impedance spectroscopy (EIS) measures the response of an electrochemical cell to an applied sinusoidal voltage signal of small amplitude as a function of the frequency. EIS is a very powerful technique for characterizing electrode processes occurring at the working electrode. Based on analyzing the complex electrical resistance (impedance) ocurring at the electrode surface, EIS is very sensitive to surface phenomena and changes of bulk properties surrounding the voltammetric electrodes, *e.g.* adsorption processes [21]. The objective of EIS was to study the adsorption kinetics of BPA on carbon (µGCE electrodes) and metal (µPtEs) surfaces.

Two types of EIS measurements were carried out, namely: (i) capacitive (without mediator); and (ii) faradaic (using FcMeOH 2.5^.^10^–3^ M mediator). The purpose is to enhance changes in *C*_*dl*_ and *R*_*ct*_ due to the adsorption of the PS-MPs in (i) and (ii), respectively.

**Fig. 3:**
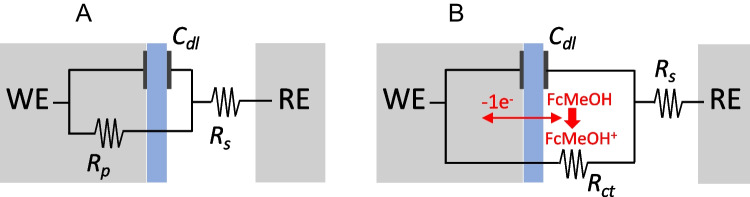
Equivalent circuits used for the fittings of the adsorbed polystyrene layers: **A** a series of capacitance and resistance in capacitative measurements (RC); **B** a parallel circuit in faradaic mode (R[CR])

A simple serial capacitance and resistance equivalent circuit (RC) was used for (i) capacitive (nonfaradaic) EIS measurements (Fig. 3A). The objective was to measure changes (decreases) in the electrode capacitance in the absence of redox reactions due to adsorption of the PS-MPs.

The standard Randles equivalent circuit R(CR) of Fig. 3B was the one that provided the most accurate fit in the (ii) faradaic measurements with the μGCn electrode (i.e., it is the most appropriate theoretical equivalent circuit to the physical processes of the experimental design). The typical diffusional mass transfer in the Randles circuit (see Fig. SI-5B), namely the Warburg impedance, can be neglected owing to the large concentration of redox mediator species and the low currents in the microelectrodes. This means that magnitudes of the impedance component due to the limiting diffusion transfer measured at low frequencies are very low. Only one semicircle appeared in the Nyquist plots in studying the adsorption of the PS-MPs (Fig. SI-5B), which signify only one RC time constant in the range of frequencies measured. As for example, fittings of the measured impedance data taking into account the Warburg impedance (R(C[RW]), see the equivalent circuit of the Fig. SI-5A)) or an additional layer interfase due to the adsorbed layer of the PS-MPs on the µGCE (as in R(C[R(RC)]) produced worse results than the simple R(CR) circuit of the Fig. 3B. Consequently, the overall impedance under the measured experimental conditions corresponds to the simplified circuit of Fig. 3B. Typical data of these fittings are set out in the supporting information (Table SI-2).

**Fig. 4:**
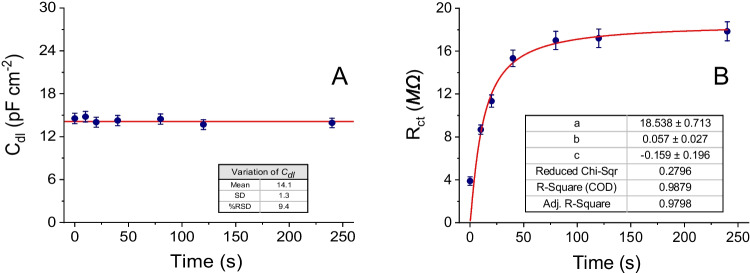
EIS measurements of **A** C_dl_ (without mediator); and **B** R_ct_ (with charge-transfer mediator, [FcMeOH] = 1^.^10^–3^ M) as a function of the adsorption time (t_ads_) of PS-MPs 0.50 pM on µGCEs. Supporting electrolyte: KCl 1^.^10^–3^ M

As shown in Fig. 4, adsorption of the PS-MPs did not produce significant changes in the capatitive layer (*C*_*dl*_) of the µGCEs in capacitive EIS measurements without mediator (equivalent circuit of Fig. 3A). Probably because the surface of the electrode is not completely covered by the adsorbed layer of microplastics behaving as not a complete insulator. Nevertheless, the adsorption of PS-MPs onto µGCEs over time (*t*_*ads*_) produce significative increases of the *R*_*ct*_ of the mediator FcMeOH in faradaic EIS measurements (equivalent circuit of the Fig. 3B). In this case, the impedance due to *R*_*ct*_ contributes predominantly to the overal impedance, A complete monolayer of adsorbed PS-MPs on the µGCEs was formed in about 140 s. after contacting the dispersion of the microplastics with the working electrode, but the PS-MPs hardly adsorbed on the Pt surface of the µPtEs (Fig. SI-5, supporting information). Fittings of the *R*_*ct*_ values with time correspond to a Langmuir (monolayer adsorption) isotherm model of the PS-MPs on the GCE and Pt microelectrodes.

### Voltammetric study of the kinetics of adsorption of BPA on polystyrene microparticles

The sensitivity of the voltammetric techniques allows determination of very small concentrations of BPA and to study their adsorption on microplastics. PS-MPs can potentially adsorb and concentrate harmful organic molecules causing consequently a synergic pollution of the ecosystems. The purpose of this part of the work is the study of the kinetics and efficiency of the adsorption of BPA on PS-MPs. Dispersions with different amounts of PS-MPs were mixed with 1.2^.^10^–4^ M of BPA and kept under stirring. At different times of adsorption (*t*_*ads*_), the separation of the PS-MPs from the solution was carried out by centrifugation, and the concentration of BPA in the solution was quantified with DPV, as described.

From the concentration of BPA in the solution and the initial amount of BPA added, the amount of BPA adsorbed on the MPs, the percentage of BPA adsorbed (*%EA*) and the amount of BPA adsorbed per unit mass of the MPs (adsorption capacity) were calculated in function of time [4]. For these calculations the following equations were used:

1 $$\%AE=\frac{C_{\mathit0}-C_e}{C_{\mathit0}}\times100$$

2 $$Q_A=\frac{C_{\mathit0}-C_e}m\times V$$

where *Q*_*A*_ is the amount of BPA that is adsorbed per gram (mass unit) of MPs (mg g^−1^); *C*_*0*_ and *C*_*e*_ refer to the initial and equilibrium concentrations of BPA in solution (mg L^−1^), respectively; *m* is the mass of the MPs (g); and *V* is the volume in which the adsorption of BPA (L) volume in which the BPA is adsorbed when in contact with the MPs.

The adsorption efficiency (*%AE*) means the percentage of BPA that remains adsorbed after the time of being in contact under vertical stirring. The adsorption capacity (*Q*_*A*_, mg g^−1^) signifies the mg of BPA retained per g of the PS-MPs, and was calculated in the time in which the highest *%AE* value was observed (typically 24 h.). Linear calibrations were also measured in BPA solutions subjected to the same centrifugation process used for the separation of the MPs (see Fig. SI-8). No significant changes were observed in the calibration equations with centrifuged and not-centrifuged BPA solutions.

Adsorption isotherm equations are usually applied to demonstrate interactions between adsorbate and adsorbent at equilibrium. From the *%AE* data as a function of time, the results were fitted to two types of adsorption models by using the following equations:

- A Langmuir isotherm (more suitable for the formation of a single adsorbed monolayer on the surface of the MPs):
  3 $$F(x)=\frac{a\cdot b\cdot x^{1-c}}{1+b\cdot x^{1-c}}$$
- A Freundlich isotherm (best suited to adsorption/retention of completed BPA multilayers on the MPs) in a standard (4), extended (5), and abbreviated (6) forms:
  4 $$F(x)=\frac xm=k\cdot x^{1/n}$$
  5 $$F(x)=a\cdot x^{{b\cdot x}^{-c}}$$
  6 $$F(x)=a\cdot x^b$$

The *%AE* results were fitted to the corresponding equations with OriginPro 2020 software. After optimizations, *%AE* data vs time was fitted to the Langmuir model (3) and the abbreviated Freundlich model (6), given the standard (4) and extended (5) Freundlich models did not converge adequately. The nonlinear curve fittings were evaluated taking into account the adjusted R-square (coefficient of determination taking into account the number of predictors in the fitted line), the residual sum of the squares, and the reduced chi-square (scale error with square, equal to the residual sum of the square of the vertical deviations divided by the degree of freedom) of the two functions.

Table 1 summarizes the values of *Q*_*A*_ with several dosages (0.2 to 1.6 g L^−1^) of polystyrene MPs. PS and EPS microparticles were prepared as described in the experimental section and size-characterized by optical microscopy (Fig. SI-9). The amount of BPA adsorbed on the microplastics did not increase significantly after a *t*_*ads*_ of 24,0 h., so the *Q*_*A*_ magnitudes were calculated with the *%AE* values at this time.

**Table 1:** Bisphenol A retention capacity (QA, mg BPA g^−1^ MPs) as a function of the amounts of polystyrene MPs in suspension for an adsorption equilibrium time of t_ads_ = 24 h. Data of Q_A_ are the mean ± sd of *n* = 3 independent measurements

MPs concentration (g L^−1^)	Kind of microplastic	*Q*_*A*_ (mg g^−1^)
0.2	PS-MPs	5.66 ± 0.28
0.4		3.10 ± 0.16
0.8		1.60 ± 0.09
1.6		0.84 ± 0.05
0.2	EPS-MPs	5.48 ± 0.27
0.4		2.87 ± 0.16
0.8		1.51 ± 0.09
1.6		0.78 ± 0.04

**Fig. 5:**
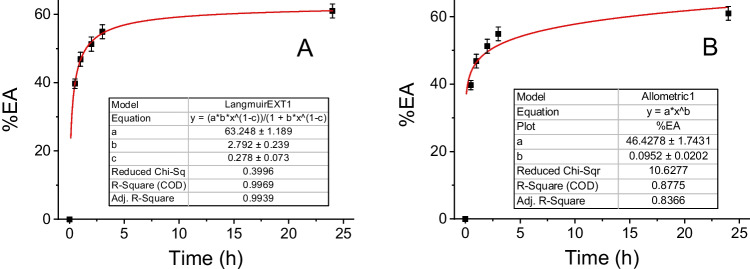
Representative plots of the adsorption efficiency (%EA) in function of the adsorption time (tads), for a dosage of 0.4 mg mL^−1^ of PS-MPs (0.50 µm diameter). Fittings to a Langmuir (**A**) and Freundlich (**B**) isotherm adsorption models. Concentration of BPA: 1^.^10^–4^ M

Figure 5 shows representative plots of the adsorption efficiency in function of time for a dosage of 0.4 mg mL^−1^ of EPS fitted to a Langmuir (3) and Freundlich (5) isotherm adsorption models. All the resulted measurements and plots are summarized in the supporting information (Fig. SI-10).

As consequence of the results, a better modeling of the BPA adsorption on the MPs is observed with a monolayer model in both PS and EPS microplastics, which means not excessively strong retention forces. The calculation of *Q*_*A*_ for an equilibrium situation (24-h adsorption time) allows us to evaluate the maximum amount of BPA that can be retained by both microplastics in the experimental conditions used.

The results are summarized in Table 1. The most important conclusion is that greater amounts of dispersed microplastics (from 0.2 to 1.6 g L^−1^) produce significant decreasings of the retention capacity of the polystyrene (from about 5.5 to 0.8 mg BPA g^−1^ (E)PS-MPs). This conclusion agrees with that found by other authors in similar studies of BPA adsorption on polyvinyl chloride microplastics [4]. This behavior may be due to the aggregation of the plastic microparticles themselves under the experimental conditions used, which causes their adsorption capacity to decrease significantly because decreasing their area per unit volume ratio.

## Conclusions

The individual irreversible adsorption events of PS-MPs from very diluted dispersions can be monitored by the blocking of the charge transfer of a FcMeOH mediator at the electrode surface. The recorded current in chronoamperometry decreased in a stpewise manner owing to these individual collision events. The magnitude of the current steps (*∆I*_*s*_) is proportional to the square of the size of the PS-MPs (${r}_{p}^{2}$) which can be used for measuring size distributions of the PS-MPs. The linear relationship between the frequency of the collision events with the PS-MPs in the range 0.005–0.500 pM also allows to measure the number concentrations under a metrological point of view. A very low concentration of supporting electrolyte (KCl 1^.^10^–3^ M) increases the flux mass transfer of the negatively charged PS-MPs to the electrode by electrophoretic migration, enhancing the sensitivity of the PS-MPs quantifications.

Faradaic EIS confirms the adsorption of the PS-MPs on GC microelectrodes because of significant changes of the charge-transfer resistances of the FcMeOH mediator in the same experimental conditions as in the blocking electrochemistry measurements. Nevertheless, capacitive changes of the double layer are not related with the irreversible adsorption of the microplastics on the working electrode. PS-MPs rapidly adsorbs on the µGCEs in a domain time of about 3–4 min of being in contact, and in a lesser extent on µPtEs.

Sensitive DPV quantification of BPA in solution allows to study the adsorption of bisphenol A on polystyrene microparticles. The proposed method can be easily adapted to other electroactive pollutants (albeit with the limitation of their electroactive nature) and on other microplastics. The adsorption kinetics was modeled to a monolayer (expanded Langmuir isotherm equation) and to a multilayer (abbreviated Freundlich isotherm equation) models. The best fits were obtained with the Langmuir model, suggesting weak adsorption forces. The maximum retention capacities of the BPA on the PS-MPs were approximately of the order of 5.5 mg BPA g^−1^ MPs, decreasing as the amount of MPs in dispersion increased (g L^−1^). This behavior was due to the aggregation of the PS-MPs during the time the measurements were taken, which makes it decrease their area/volume ratio and its ability to retain BPA.

## Supplementary information

Below is the link to the electronic supplementary material.

Supplementary file1 (DOCX 2.29 MB)
