# Supplementary material for: Detection, quantification, and characterization of polystyrene microplastics and adsorbed bisphenol A contaminant using electroanalytical techniques
Source: Mikrochim Acta. 2023 May 9;190(6):203. doi: 10.1007/s00604-023-05780-5 (PMC10167125; doi:10.1007/s00604-023-05780-5)
Supplement: Supplementary file 1 — Supplementary file1 (DOCX 2.29 MB) [file 604_2023_5780_MOESM1_ESM.docx]

**Supporting Information**

Electronic Supporting Material on the Microchimica Acta publication entitled

Detection, quantification and characterization of polystyrene microplastics and adsorbed bisphenol A contaminant using electroanalytical techniques

Juan C. Vidal,^*^ Javier Midon, Ana Belén, Dragos Ciomada, Francisco Laborda

Analytical Spectroscopy and Sensors Group (GEAS), Institute of Environmental Sciences (IUCA), University of Zaragoza, c/ Pedro Cerbuna 12, 50009-Zaragoza, Spain.

** Corresponding author. E-mail: jcvidal@unizar.es*

**Table of Contents**

**Instrumentation**.

Electrochemical characterization of the microelectrodes (µGCEs, µPtEs) 2

**Results and Discussion.**

Evaluation of the diameter of the PS-MPs by the blocking collision electrochemistry 3

Influence of the working electrode substrate in the blocking electrochemistry of the PS-MPs 4

Quantification of plastic microparticles by the blocking electrochemistry procedure 4

Electrochemical Impedance Spectroscopy of the plastic microparticles with Pt microelectrodes 5

Quantification of bisphenol A by differential-pulse voltammetry 7

Kinetics of the adsorption of BPA on PS-MPs as a function of the microplastics dossage 8

**References.** 9

**Instrumentation**

**Electrochemical characterization of the microelectrodes (µGCEs, µPtEs)**

The working microelectrodes (µGCEs, µPtEs) were size-characterized for the correct interpretation of the results and comparisons among different electrodes. Cyclic voltammograms of a 0.5^.^10^-3^ M of FcMeOH in 0.10 M. KCl solution were recorded at scan rates from 20 to 200 mV s^-1^ (Fig. SI-1). The calculation of the real area of the electrodes was carried out by cyclic voltammetry, based on the theoretical equation Randles–Sevcik (1):

*i_p_* = 2.69 ^.^ 10^5^ ^.^ *n*^3/2^ ^.^ *v*^1/2^ ^.^ *D*^1/2^ ^.^ *A* ^.^ *C*

where *i_p_* is the (anodic) voltammetric current obtained (A), *n* is the number of electrons in the electrochemical reaction, *v* is the potential scan-rate (V s^-1^), *D* is the diffusion coefficient of FcMeOH (1^.^10^-5^ cm^2^ s^-1^), *A* is the electroactive area of the working electrode (cm^2^) and *C* the concentration of FcMeOH (mol cm^-3^).

**Fig. SI-1**. Cyclic voltammograms of a FcMeOH 0.5^.^10^-3^ M in KCl 0.10 M solution at scan rates of (a) 200 mV s^-1^; (b) 100 mV s^-1^; (c) 50 mV s^-1^; and (d) 20 mV s^-1^ on GC and Pt microelectrodes.

Mass transport in the microelectrodes is dominated by radial or spherical diffusion and the current reaches a steady state value independent of the time domain (that is, on the potential scan rate), given that a sufficient concentration gradient is not produced to limit the total current by diffusion.

The mean nominal geometric areas of the microelectrodes are 95.0 μm^2^ for the μGCE (11±2 μm diameter) and 78.5 μm^2^ for the μPtE (10±2 μm diameter). The active area was calculated from the slope of the calibration plot of peak current versus the square root of the scan rate, by applying the values from the equation and the uncertainty of the calibration. The results are summarized in Table SI-1 for the four electrodes used. The effective areas were slightly higher than the geometrical area. The real areas were used to calculate the current and charge densities of the electrochemical measurements.

| Microelectrode | *Active area, μm^2^ | *Diameter, μm |
| --- | --- | --- |
| μGCE - 1 | 106 ± 2 | 12 ± 1 |
| μGCE - 2 | 106 ± 3 | 12 ± 1 |
| μPtE - 1 | 85 ± 4 | 10 ± 1 |
| μPtE - 2 | 93 ± 4 | 10 ± 1 |

** Mean ± uncertainty of the linear regression*

**Table SI-1**. Active areas and diameters of the microelectrodes used, calculated from the cyclic voltammograms of a solution FcMeOH 0.5^.^10^-3^ M in KCl 0.10 M by using the Randles-Sevcik equation.

**Results and Discussion**

**Calculation of the diameter of the PS-MPs by the blocking collision electrochemistry**

Remarkably, the magnitudes of *∆I_s_* are related to the square of the radius of the plastic microparticles that produce the blocking collisions. This allows obtaining size distributions of the PS-MPs, which can be calibrated by using size standards and under the same experimental conditions (mainly the concentration of the mediator and the supporting electrolyte). As an example, Fig. SI-2 shows distributions of *∆I_s_* from PS-MPs with nominal diameters of 0.50 and 0.10 µm.

Fig. SI-2. Histograms of current step heights (∆Is) for the adsorption of PS-MPs (0.05 pM) with nominal diameters of: (A) 0.50 μm; and (B) 0.10 μm. Solution: 2.5^.^10^-3^ M of FcMeOH in KCl 1^.^10^-3^ M. Working electrode: µGCE.

This confirms the relationships between *∆I_s_* and $r_{p}^{2}$. In this example, the ratio between the two means of both distributions has a value very close to the ratio of both $r_{p}^{2}$.

As reported by other authors, the wide variation in the current steps (high standard deviations of the histograms) can be explained by different landing positions of particles on the electrode surface. Particles landing closer to the edge of an electrode disk result in bigger steps because they block the flux of the redox species to a larger extent than those particles landing closer to the disk center [ (2)].

**Influence of the working electrode substrate in the blocking electrochemistry of the PS-MPs**

Platinum working microelectrodes measured lower current step magnitudes than glassy-carbon microelectrodes under the same experimental conditions. This is illustrated in Fig. SI-3. This can be explained by the lower charge transfer rate of FcMeOH and the lower adsorption capacity of PS-MPs on Pt surfaces compared to GC surfaces.

Fig. SI-3. Distribution of current step magnitudes (∆I_s_) measured with a μPtE from a solution PS-MPs 0.05 pM (0.50 μm diameter). Experimental conditions as in Fig. 2A of the main text.

**Quantification of plastic microparticles by the blocking electrochemistry procedure**

The frequency of the current steps measured in the chronoamperograms (from 10 to 130 s.) is related to the number of the plastic MPs. This allows the analytical quantification of the polystyrene microparticles. Fig. SI-4 shows a typical calibration in which a linear relationship was measured in a range 0.005-0.500 pM of PS-MPs.

Fig. SI-4. Calibration plot of the PS-MPs. There is a linear relationship between the count of the current steps and the number concentration of the PS-MPs.

**Electrochemical Impedance Spectroscopy of the plastic microparticles with Pt microelectrodes**

Fig. SI-5 shows an equivalent Randles circuit taking into account a Warburg impedance (A) and the corresponding Nyquist plot (B). In a Randles circuit (R(C[RW]) is represented the resistance of the electrolyte (*R_s_*), the capacitance of the electrode double-layer (*C_dl_*), the resistance of the mediator charge-transfer (*R_ct_*) and the Warburg impedance (*Z_W_*). Warburg impedance prevails at low frequencies over *R_ct_*.

Fig. SI-5B represents a typical Nyquist plot from the Randles circuit R(C[RW]) of the Fig. SI-5A. Z´´and Z´are the imaginary numbers used to represent the impedance, and the with of the semicircle represents the *R_ct_* magnitude. Zw corresponds to the 45° line at low frequencies.

Fig. SI-5. (A) EIS equivalent Randles circuit with limiting diffusional transfer Warburg impedance (R(C[RW])). (B) A typical Nyquist plot corresponding to one RC parallel equivalent circuit with a diffusion limited process. The diameter of the Nyquist plot represents the ohmic resistance of the charge-transfer of the mediator (R_ct_) in the EIS faradaic measurement.

The Warburg impedance can be neglected in the experimental conditions of this study owing to the large concentration of the FcMeOH mediator and the low currents (*i.e.*, negiglible concentration gradients) measured with the microelectrodes.

The adsorption of the PS-MPs on the Pt and GC microelectrodes produced changes in the charge-transfer resistance (*R_ct_*) of the mediator FcMeOH measured by EIS in faradaic mode, as explained in the main text (equivalent circuit of the Fig. 3B). However, negigible changes were measured in *C_dl_* magnitudes (EIS capacitive mode) due to the adsorption of the PS-MPs on both microelectrodes.

Representative data of the EIS measurements calculated from the equivalent circuits of the Fig. 3B are given in Table SI-2. Attempts to add an additional Warburg impedance component gave magnitudes in the range *Y_0_*≈32.1-22.8 nMho**^.^**s^1/2^, but with irreproducible results owing to incorrect fittings.

|  | μGCE | | | μPtE | | |
| --- | --- | --- | --- | --- | --- | --- |
| *t* (s) | *R_s_* (kΩ) | *C_dl_* (pF cm^-2^) | *R_ct_* (MΩ) | *R_s_* (kΩ) | *C_dl_* (pF cm^-2^) | *R_ct_* (MΩ) |
| 0 | 55.80 ± 2.73 | 15 ± 0.6 | - - | 50.60 ± 2.56 | 14 ± 0.6 | - - |
| 10 | 51.18 ± 2.50 | 15 ± 0.6 | 8.64 ± 0.45 | 56.39 ± 2.84 | 14 ± 0.6 | 26.10 ± 1.58 |
| 20 | 49.63 ± 2.43 | 14 ± 0.6 | 11.31 ± 0.58 | 49.66 ± 2.50 | 14 ± 0.6 | 27.01 ± 1.64 |
| 40 | 59.94 ± 2.92 | 14 ± 0.6 | 15.37 ± 0.78 | 42.88 ± 2.16 | 13 ± 0.6 | 28.63 ± 1.72 |
| 80 | 48.55 ± 2.39 | 14 ± 0.6 | 17.03 ± 0.86 | 43.86 ± 2.21 | 13 ± 0.6 | 28.82 ± 1.75 |
| 120 | 56.39 ± 2.75 | 14 ± 0.6 | 17.32 ± 0.88 | 39.22 ± 1.98 | 12 ± 0.5 | 28.98 ± 1.72 |
| 240 | 60.94 ± 2.99 | 13 ± 0.5 | 17.83 ± 0.89 | 47.41 ± 2.38 | 12 ± 0.5 | 30.02 ± 1.82 |

Table SI-2. Representative data fitted from the EIS equivalent circuits of the Fig. 3B, measured by the μGCE and μPtE microelectrodes. Data are the mean ± sd of n=4 independent EIS measurements.

The adsorption of PS-MPs on µPtEs produced lesser changes in *R_ct_* than on µGCEs in EIS faradaic measurements, as it is shown in Fig. SI-6B and comparing with Fig. 4B. Negiglible changes in *C_dl_* magnitudes were measured in capacitive mode with the µPtEs (Fig. SI-6A), the same behaviour that with µGCEs.

Fig. SI-6. Measurements of C_dl_ and R_ct_ as a function of the adsorption time of PS-MPs on a μPtE microelectrode. Experimental conditions as in Fig. 4 (main paper).

**Quantification of BPA by differential-pulse voltammetry**

Typical voltammograms in the DPV voltammetric determination of BPA, by using conventional GCEs, are shown in Fig. SI-7. Calibration graphs of BPA are depicted in Fig. SI-8, comparing both signals peak-height currents and areas of the voltammetric peaks.

Fig. SI-7. Representative differential-pulse voltammograms of: (A) Blank Solution; (B) BPA 10.0 µM. Supporting electrolyte: KCl 0.10 M. Working electrodes: glassy-carbon electrodes.

Fig. SI-8. Calibration plots of the DPV voltammetric determination of BPA. Supporting electrolyte: KCl 0.10 M. Working electrode: μGCE. Comparison between measurements of the maximum peak-height current (i_p_) and the area of the peak (Q_p_).

The parameters of the linear regression equations are given in Fig. SI-8.

Optimization of the DPV voltammetric determination with *bare* GCE electrodes gave the following analytical properties:

(A) Peak current signals (*i_p_*, µA):

Linear range: 0.80-15.00 µM; Reproducibility (for [BPA]=5^.^10 µM): 4.57% (%RSD); Limit of detection (3^.^SD)= 0.24 µM

(B) Peak area signals:

Linear range: 1.40-15.00 µM; Reproducibility (for [BPA]=5^.^10 µM): 4.16% (%RSD); Limit of detection (3^.^SD)= 0.42 µM

**Kinetics of the adsorption of BPA on PS-MPs as a function of the microplastics dossage**

To study the adsorption of BPA on microplastics, PS and EPS samples were prepared as described in the main paper. These samples were characterized by optical microscopy (Fig. SI-7), obtaining size distributions of 48.6 ± 9.8 µm (PS-MPs) and 65.5 ± 13.1 µm (EPS-MPs) (mean ± sd). The number of particles measured was greater than 80 in both cases.

Fig. SI-9. Photomicrographs of polystyrene particles: (A) PS 50x magnification; (B) PS 100x magnification; (C) EPS 50x magnification; and (D) EPS 100x magnification.

­­Fig. SI-10 shows the nonlinear fittings of the adsorption isotherms of BPA on PS-MPs and EPS-MPs at 0.4 and 1.6 g L^-1^ microplastic dossages.

Fig. SI-10. Adsortion kinetics of BPA on PS microplastics as a function of the microplastic dossage. Nonlinear fittings to a Langmuir and Freundlich (Allometric1 function) models.

**References**

1. Vidal JC, Torrero D, Menés S, de La Fuente A, Castillo JR (2020) Voltammetric sensing of silver nanoparticles on electrodes modified with selective ligands by using covalent and electropolymerization procedures. Discrimination between silver(I) and metallic silver. Microchimica Acta 87:183-194. <https://doi.org/10.1007/s00604-020-4139-5>.

2. Boika A, Thorgaard SN, Bard AJ (2013) Monitoring the Electrophoretic Migration and Adsorption of Single Insulating Nanoparticles at Ultramicroelectrodes. J. Phys. Chem. B 117:4371-4380. <https://doi.org/10.1021/jp306934g>.
